# Advances in understanding migraine pathophysiology: a bench to bedside review of research insights and therapeutics

**DOI:** 10.3389/fnmol.2024.1355281

**Published:** 2024-02-28

**Authors:** Kofi Frimpong-Manson, Yuma T. Ortiz, Lance R. McMahon, Jenny L. Wilkerson

**Affiliations:** Department of Pharmaceutical Sciences, Texas Tech University Health Sciences Center, Amarillo, TX, United States

**Keywords:** serotonin, calcitonin gene related peptide (CGRP), cannabinoid, rodent, cortical spreading depression, purinergic receptor, nitroglycerin, addiction

## Abstract

The individual and global burden of migraine is of such significance that there are accelerated efforts to develop new therapies. New migraine therapeutics are needed to address the current deficiencies that exist in the efficacy and adherence rate of approved anti-migraine medications. The recent discovery of the calcitonin gene related peptide as an add-on to the role of serotonin has markedly increased the range of new treatment options for acute and chronic migraine. Despite this, tackling the complexity of migraine disorders requires a complete understanding of its pathophysiology. Preclinical animal models can shed light on disease-related pathophysiology, including migraine. Indeed, the use of animal models has been instrumental in developing many therapeutics. However, an animal model is limited by the predictive and face validity of that model, and this extends to preclinical migraine models. In this review, a summary of the current understanding of the pathophysiology of migraine is given from both a preclinical and clinical perspective, and an emphasis is placed on the animal models of migraine. We will discuss the strengths and pitfalls of common preclinical migraine models as well as experimental research areas to explore further.

## 1 Introduction

Migraine and associated symptoms are a significant burden to the financial, mental, and physical wellbeing of those that live with this chronic disease. A retrospective study conducted in the United States showed that migraine patients spent as much as $11,010 annually in direct healthcare costs on average in an attempt to treat their disease which included frequent emergency room, inpatient and outpatient visits and medication prescriptions (Bonafede et al., 2018). In the same study, the annual indirect costs of migraine patients due to disability and workplace absenteeism were about $2350 higher as compared to matched non-migraine patients. In a survey conducted on about 160,000 Americans aged 12 years and above in 2012 under the American Migraine Prevalence and Prevention Study (AMPP), and in line with other studies, the prevalence of migraine was higher in females compared to males across all age groups. A total 7.68% of the study population had migraine and within this group 0.91% were chronic migraine patients (Buse et al., 2012). Chronic migraine was also associated with the highest headache related disability. It is important to note the observation that within this study chronic migraine prevalence was higher in low-income households. Although it has been reported that high income developed countries have statistically higher migraine populations than poorer countries, this may be due to the point that more studies are conducted in high income countries which could skew data (Stovner et al., 2022). Migraine has a sex-related pattern as well and with a global prevalence of 11.24 and 19.01%, respectively, for males and females using data obtained from 204 countries and territories in 2019 (GBD 2019; Diseases and Injuries Collaborators, 2020). A clinically observed sex-related difference is important to consider in drug discovery research due to the implications related to chronic migraine preclinical model validation. Despite significant financial, mental and physical burdens, a need persists for a better understanding of chronic migraine neurobiology, underlying receptor mechanisms, as well as therapeutic treatment options.

The understanding of migraine pathophysiology has evolved over the years. There are several different theories surrounding migraine development and maintenance. These theories include: the vascular and the neurovascular theories of migraine (Graham, 1938; Goadsby et al., 1990). Each of these theories will be discussed in this review. One of the earliest theories postulated was the vascular theory. A study observed that ergotamine tartrate, through its vasoconstrictive effects, was able to reduce the pulsations as well as headache associated with migraine (Graham, 1938). The importance of vasodilation in migraine pathophysiology has been further investigated by observing changes that occur in blood vessels within the brain. It was initially hypothesized that dilation of the temporal and middle meningeal arteries might play a role (Graham, 1938). Although it was observed that migraine relief could be produced after compressing the temporal artery, it was shown to occur in only few migraine patients (Drummond and Lance, 1983). The external carotid artery has also been implicated to be involved in migraine headache (Ray and Wolff, 1940). The vascular theory has remained a controversy due to emerging evidence that refutes this explanation, including the finding that not all vasodilators cause migraine. In a randomized clinical trial that involved the infusion of vasoactive intestinal peptide (VIP) into 12 patients, migraine-like headache was not triggered in migraine patients, although there was vasodilation of the superficial temporal artery (Rahmann et al., 2008). In another study that involved the use of adrenomedullin to induce migraine headache, despite a vasodilatory response seen in blood vessels, there was no difference compared to placebo (Petersen et al., 2009) and these findings have been replicated in a preclinical study involving a mouse migraine model (De Logu et al., 2019). However, it is still recognized that vasodilation is an important mechanism that contributes to migraine and some current human and animal models of migraine still rely on vasodilatory properties of migraine headache inducers to assess novel compounds. Critical unknown migraine pathophysiological areas that are currently of interest in the migraine research community include: determining an initial trigger for migraine, the role of central vs. peripheral migraine headache mediation, the contributing factors that lead to either an acute or sustained migraine attack, as well as biomarkers that correlate with migraine symptoms.

Currently, the prevailing theory embraces the role of inflammation and neural release of peptides in the development of migraine headache in addition to the vascular theory, and migraine is thus considered to be a neurovascular condition. This review will provide a cursory overview of the dominant processes that contribute to migraine pathophysiology. It will also cover the associated clinical features of migraine, human and animal models of migraine with their strengths and weaknesses, migraine therapeutics as well as areas that could be considered for future research. Moreover, a major emphasis will be placed on preclinical migraine models and experimental therapeutic development of antimigraine drugs. Although important to migraine and associated symptomology, this review will not cover *in vitro* models of migraine, other migraine types including acute and chronic migraine with or without aura, non-pharmacological treatments, and in-depth mechanisms of action for the different treatment options. For further review please see the following references on pathophysiology of migraine (Goadsby et al., 2017), experimental models of migraine (Harriott et al., 2019), and treatment options for migraine (Zobdeh et al., 2021).

The literature search was conducted using PubMed, Web of Science and SCOPUS using the search words: migraine, cortical spread depression, migraine pathophysiology, and migraine treatment. The definitions of migraine are limited to that provided by the International Headache Society (Headache Classification Committee of the International Headache Society (IHS), 2018). In databases that allowed filtering search results, the search was limited to original research articles. Original research articles were screened to identify experimental work carried out on rodents while clinical research papers were limited to randomized clinical trials and meta-analysis. All research included in this review needed to fulfill the requirement of appropriate treatment and control groups with objective and unbiased conclusions. Research papers that utilized non-pharmacological interventions were excluded.

### 1.1 What is migraine?

In discussing migraine, it is imperative to distinguish an acute migraine from other headache classes, as well as chronic migraine from chronic headaches. Headache is a subjective symptom which is commonly reported among patients and ranges in severity from mild to severe. Its impact on health is so profound that it ranked in 2019 according to the Global Burden of Disease Study among the top ten conditions for disability-adjusted life-years (DALYs). DALYs are a measure of the number of years lost due to poor health, disability or early death to express disease burden (GBD 2019; Diseases and Injuries Collaborators, 2020). To facilitate diagnosing criteria for different types of headache, the International Headache Society (IHS) is responsible for publishing the International Classification of Headache Disorders and their third edition was published in 2018 based on emerging scientific evidence on headaches (Headache Classification Committee of the International Headache Society (IHS), 2018). Primary headaches are headaches without an underlying cause, and migraine in addition to tension-type headache are the two most common primary headache disorders with a quantifiable impact on global population health (Steiner and Stovner, 2023). It is also worth noting that migraines have been shown to be the second highest cause of disability among all populations globally using age-standardized years of life lived with disability (YLD) as a metric (Stovner et al., 2018; Steiner and Stovner, 2023). The loss of productivity associated with migraines is evident since the group most affected form the largest proportion of the workforce that drives national economies. The migraine disability assessment (MIDAS) questionnaire is a 5-point validated questionnaire that is also used to grade migraine associated disability using a scoring system (Stewart et al., 2000, 2001). According to the MIDAS tool, disability associated with migraine is described as the number of days missed at work or school, reduced productivity at home, work, and school as well as reduced social activities within 3 months (Stewart et al., 2000, 2001). After summing the number of days associated with headache/migraine-related disability, the total obtained is used to categorize severity into grades as shown in Table 1.

**TABLE 1:** MIDAS questionnaire for migraine disability (Stewart et al., 2001; Ford et al., 2020).

1. On how many days in the past 3 months did you miss work or school because of your headaches?		
2. How many days in the past 3 months was your productivity at work or school reduced by half or more because of your headaches?		
3. On how many days in the past 3 months did you not do household work because of your headaches?		
4. How many days in the past 3 months was your productivity in household work reduced by half or more because of your headaches?		
5. On how many days in the past 3 months did you miss family, social or non-work activities because of your headaches?		
A. On how many days in the past 3 months did you have a headache?		
B. On a scale of 0-10, how painful were these headaches on average?		
Grade I	Little or no disability	0–5
Grade II	Mild disability	6-10
Grade III	Moderate disability	11-20
Grade IV-A	Severe disability	21-40
Grade IV-B	Very severe disability	>40

Acute migraine is described as headache that occurs unilaterally with a pulsating sensation, which lasts between 4 to 72 h and is associated with nausea and/or light sensitivity (photophobia) and sound sensitivity (phonophobia) (Headache Classification Committee of the International Headache Society (IHS), 2018). This headache class may be of moderate to severe intensity and may be exacerbated by physical activity. In some instances, the headache may be described as pressure, stabbing or aching (Kelman and Tanis, 2006). A patient who experiences headache, but not necessarily a migraine, on 15 or more days within a month for more than 3 months, with features of migraine headache on at least 8 days per month, can be said to have chronic migraine based on the ICHD-3 (Headache Classification Committee of the International Headache Society (IHS), 2018). Importantly, a challenge that these diagnostic criteria poses is the reliance on the patient’s ability to recall number of headache days accurately. It has been argued that the current definition of chronic migraine be reconsidered, and the threshold of days lowered since there are patients that have about 8 to 14 headache days per month but with a similar disease burden as 15 days or above (Ishii et al., 2021). These patients that experience a lower threshold may fail to get the needed medical treatment and represents another challenge in the treatment of chronic migraine.

### 1.2 Migraine with and without aura

According to the studies, there are two types of migraine classified based on the presence or absence of aura, i.e., migraine with aura and migraine without aura (Rasmussen and Olesen, 1992; Headache Classification Committee of the International Headache Society (IHS), 2018). An aura describes reversible neurological symptoms that have a short duration and may manifest before a migraine headache or occur concurrently with the headache. These may include one or more of the following symptoms: visual (an absolute or partial blind spot at a fixed point with zigzag pattern, which has a distorted and bright edge of convex shape), sensory disturbances (numbness, migratory pins and needles sensation on body, face and/or tongue), speech disturbance (aphasia characterized by difficulty in expressing oneself), motor weakness, brainstem symptoms (tinnitus, vertigo/dizziness, diplopia/double vision, dysarthria/difficulty speaking, decreased consciousness).

Other classifications of migraine exist which are based on their unique clinical presentations which may differ in onset, frequency, or associated symptoms. For instance, hemiplegic migraine describes migraine with associated motor weakness, while migrainous infarction is a type of migraine that co-occurs with ischemic infarctions in the brain. A detailed list of these classifications is given by the International Headache Society in the International Classification of Headaches, 3rd Edition and are out of the scope of this review.

### 1.3 Symptoms associated with migraine

There are some patients who experience a prodromal phase (also referred to as premonitory phase) that heralds an impending migraine headache (Giffin et al., 2003) and may start hours or a few days to the attack; it may manifest as tiredness, photophobia and/or phonophobia, or nausea. Yawning was shown to be a common prodromal symptom in a cross-sectional study of 2,714 patients conducted by Laurell et al. (2016). It must be noted that this prodromal phase is different from an aura since it does not involve neurological symptoms. A post-dromal phase has also been described and occurs following headache resolution. But it can last as long as 48 h and some of its symptoms include fatigue, difficulty in concentration and stiffness of the neck (Headache Classification Committee of the International Headache Society (IHS), 2018). In a study of 11,388 migraine patients, cutaneous allodynia was reported among about 63% of patients (Lipton et al., 2008). Cutaneous allodynia tends to be frequently associated with migraine and may occur with or without migraine headaches and some unilateral autonomic symptoms such as nasal congestion, lacrimation and eyelid edema which are associated with trigeminal autonomic cephalalgias have also been reported among migraine patients (Barbanti et al., 2002). Other symptoms reported include diarrhea, taste abnormality, osmophobia (Kelman and Tanis, 2006).

### 1.4 Migraine risk factors

#### 1.4.1 Obesity and depression

Obesity has been shown to be a risk factor for the development of migraine (Santos et al., 2015; Gelaye et al., 2017; Miri et al., 2018) and it is hypothesized that increased levels of inflammatory mediators, increased sympathetic activity, and increased leptin (Pisanu et al., 2017) which are associated with obesity might influence the development of migraine. Adiponectin is also suspected to play a role (Duarte et al., 2014; Abbasi et al., 2019; Joshi et al., 2020) although there are contrasting opinions on this (Dearborn et al., 2014; Peterlin et al., 2016). Depression is another risk factor for migraine development (Breslau et al., 2003; Karakurum et al., 2004) as well as episodic to chronic migraine progression. An episodic migraine is migraine that does not occur as frequently as occurs in chronic migraine i.e., less than 15 days per month. Results from the observational cohort study of the AMPP study showed patients who experienced episodic migraine with depression were at a higher risk of chronic migraine onset and this could be explained by genetic predispositions or environmental factors such as life events or stress that have an influence on both conditions (Ashina et al., 2012).

#### 1.4.2 Miscellaneous risk factors

One unexplained risk factor is genetic susceptibility to raised serum calcium levels. Results obtained in a Mendelian randomization study showed hereditary patterns in both migraine development as well as hypercalcemia, although the underlying mechanism that links these two conditions is unknown (Yin et al., 2017). Calcium channel blockers such as flunarizine, a non-selective calcium channel blocker that are used to prevent migraine headaches give some level of credence to the involvement of calcium ions in migraine (Li et al., 2011). Also, asthma is suspected to be a risk factor that leads to the development of chronic migraine in patients with pre-existing episodic migraine. In a study based on results of the AMPP, it was found that episodic migraine patients with migraine were twice as likely to progress to chronic migraine compared to those without asthma (Buse et al., 2012; Martin et al., 2016). It is suspected that the upregulation of inflammatory mediators in asthma may play a role in this.

### 1.5 Migraine triggers

Migraine patients have self-reported factors that trigger attacks. In a survey conducted on patients with migraine, triggers identified were classified into precipitating and aggravating factors. Some precipitating triggers which were predominant among migraine patients included weather changes, smell, smoke and light, while aggravating factors also included physical activity, noise and motion that involved straining and bending over (Spierings et al., 2001). Other triggers include stress and fatigue, lack of sleep, not eating on time, menstruation, and this has been corroborated in other studies (Ierusalimschy and Moreira Filho, 2002; Andress-Rothrock et al., 2010; Hauge et al., 2010). Recognizing the predominance of migraines in females and menstruation as a trigger, ICHD-3 has a category for menstrual migraine which is differentiated based on whether migraines occur during menstruation only, during and outside the menstruation period, or outside the menstruation period. Some foods are also an identified trigger for migraine although a mechanism remains elusive. A survey conducted in an outpatient’s neurology clinic in Turkey on 146 migraine patients showed that migraine headache was triggered by intake of chocolate, coffee, alcohol, milk and cheese although this was in a small percentage of participants (Hauge et al., 2010). In a randomized controlled trial among 38,370 females that sought to quantify the triggering of migraine on consuming some foods such as alcohol, ice cream, citrus fruits, milk and processed meat, it was found that dairy products and chocolate could be linked to the onset of migraine with aura (Rist et al., 2015). This study relied upon the consumption of low food quantities as being a marker of the food being a potential trigger for migraine, which was a limitation. It could be that the low intake of these foods was due to other factors such as general dislike for the food, and there is no evidence to suggest that food intake contributed to the migraine headache experienced.

## 2 Pathophysiology, pharmacological targets and United States Food and Drug Administration (US FDA) approved drugs for migraine

### 2.1 Pathophysiology and pharmacological targets

#### 2.1.1 Cortical spreading depression (CSD) as a trigger of migraine aura

An attempt to understand the underlying mechanisms that cause migraine aura led to the discovery of a phenomenon known as cortical spreading depression (CSD). It has been described as a slow spreading wave of depolarization in glial and neuronal cells and is seen as a slow direct current potential shift due to high extracellular potassium ions with high intracellular sodium and calcium ions (Leao, 1947). CSD has been observed in migraine with aura where the CSD threshold is lowered to facilitate triggering an aura and seemly is the pathway through which some treatments act (Ayata et al., 2006). For example, some migraine prophylactic drugs were observed to decrease chemically-induced CSD in rats with prolonged administration having a sustained negative effect on CSD (Ayata et al., 2006). One of its downstream events is trigeminovascular system activation, which facilitates the release of nitric oxide to cause vasodilation, leading to increased blood flow in the meningeal artery. In addition, neurogenic inflammatory substance release in the dura mater causes sensitization of meningeal nociceptors leading to migraine headache. Experimentally, in rats, the onset of CSD produced Fos release in the trigeminal nucleus caudalis and downstream second-order neuronal activation, which led to migraine-like vascular events (Bolay et al., 2002). This phenomenon plays such an integral role in migraine that it is also used as a tool to monitor the efficacy as well as pharmacological activity of anti-migraine drugs (Schain et al., 2019). Although CSD is a spontaneous event, it occurs under the influence of preliminary events in the brain including neuronal hyperexcitability. While CSD is linked with the initiation of migraine with aura, it does not seem to play a role in migraine without aura. It is possible that migraine without aura solely depends on vascular changes and neurogenic mechanisms that increase nociceptive receptor sensitization in the brain.

##### 2.1.1.1 Excitatory neurotransmitters and cortical spreading depression

Migraine may be linked to a state of hyperexcitability in brain activity (Ferrari et al., 1990; Petzold et al., 2008). Glutamate and aspartate are the primary excitatory neurotransmitters in the brain, and early studies involving migraine patients showed the levels of glutamate and aspartate were elevated between attacks in patients with migraine with aura as compared to controls (Ferrari et al., 1990). Between these two neurotransmitters, plasma glutamate levels have been found to be elevated over aspartate levels, with each being as high as 0.89 mg/dL and 0.167 mg/dL, respectively (Castillo et al., 1994). Because of this, glutamate has gained the most attention among the two excitatory neurotransmitters and is also seen to be equally high in migraine without aura patients (Zielman et al., 2017). In a preclinical study involving rats, it was observed that there is an increase in glutamate expression which may sensitize neurons in the trigeminal nucleus caudalis (Oshinsky and Luo, 2006). In addition, nociceptive signal transmission in the trigeminovascular system has been shown to involve N-methyl-D-aspartate (NMDA) receptors, and glutamate is an endogenous NMDA receptor agonist (Storer and Goadsby, 1999). Glutamate release and CSD have a bidirectional association in the sense that glutamate stimulates CSD, and this depolarizing wave also enhances glutamate release. In an experiment using rats, CSD was induced by potassium chloride application alone as well as in the presence of the NMDA receptor antagonist MK-801, and MK801-treated brain slices showed an increase in the threshold required for triggering CSD (Petzold et al., 2008). As already mentioned, potassium ion levels increase during CSD activation, but so do glutamate levels and the overall effect is a continuous depolarization of adjacent cells.

##### 2.1.1.2 Ions involved in cortical spreading depression

Cortical spreading depression (CSD) has been described to involve an increase in extracellular potassium levels and an increase in intracellular sodium and calcium ions (Leao, 1947; Enger et al., 2015; Cozzolino et al., 2018). Among these ions, change in potassium concentration is the most widely accepted to be implicated in CSD propagation (Tozzi et al., 2012). Grafstein (1956) demonstrated that potassium ions are released by neuronal cells to stimulate a depolarizing cascade in neuronal cells that contributes to the slow propagating wave of CSD (Grafstein, 1956; Somjen, 2001). Additionally, preclinical experiments conducted that involved the measurement of cerebral blood flow and neuronal activity after CSD induction, demonstrated that sodium channel blockers were able to inhibit CSD-induced changes in regional blood flow (Akerman et al., 2008). Topiramate is an antiepileptic with suspected sodium channel blockade activity and used to prevent migraine clinically (Silberstein et al., 2012; Sills and Rogawski, 2020). In rat neocortical slices that have been exposed to increased extracellular potassium ions to trigger CSD, topiramate reduced CSD propagation area as visualized through intrinsic optical signal imaging (Tozzi et al., 2012). The importance of calcium in CSD has also been elucidated, as L-type, N-type and P/Q-type voltage calcium channel blockers reduce repeated CSD onset (Ayata et al., 2000). In addition, mice that express P/Q type calcium channel mutations exhibit higher CSD thresholds induced by electrical and potassium chloride administration (Ayata et al., 2000). Thus, it is evident that calcium channel activation and calcium channel blockers play a critical role in migraine pathophysiology and migraine management.

As discussed above, glutamate is involved in initial CSD generation as well as propagation. Thus, the initiating events surrounding aberrant glutamate release are important in migraine pathophysiology. In a study conducted on migraine patients, magnesium levels in the brain were found to be low (Ramadan et al., 1989) and these findings have led to an interest in magnesium supplementation as part of migraine therapy. Magnesium can inhibit calcium influx, leading to decreased glutamate release and may have other roles such as inhibiting serotonin and calcitonin gene related peptide release, both of which are also contributors to migraine headache (Mathew and Panonnummal, 2021). Thus, it is suspected that low magnesium levels in the brain predispose neurons to aberrantly release glutamate. In support of this hypothesis, an experiment conducted in rats found that local but not systemic magnesium administration inhibited glutamate release as well as suppressed nociceptive responses from the trigeminal region of the brain (Hoffmann et al., 2019).

Migraine headache has long been thought to be due to vasodilation that occurs in meningeal arteries and the next section will discuss the vascular theory of migraine.

#### 2.1.2 Molecules involved in the vascular theory of migraine

##### 2.1.2.1 Nitric oxide

Nitric oxide is a potent vasodilator that has a causative role in migraine and was part of the molecules investigated to explain the vascular theory of migraine. It was demonstrated in human subjects that an infusion of nitroglycerin, a nitric oxide precursor, was associated with headache of pulsatile quality, and this administration had a ceiling effect at an infusion rate of 0.5 ug/kg/min (Iversen et al., 1989). In another clinical trial on humans, nitric oxide was shown to produce unilateral headache characteristic of migraine with an associated increase in cerebral blood flow due to vasodilation. Quantitative analysis in this study also revealed that, nitric oxide and its metabolites are released in the early phase of a migraine attack (Sarchielli et al., 2000). Not only does its external infusion trigger migraine-like headache, but it was observed that in some cases nitric oxide had the potential to trigger prodromal/premonitory symptoms of migraine (Afridi et al., 2004; Karsan et al., 2016). The action of nitric oxide is one of the major contributors to the maintenance of the vascular theory of migraine because other neuroinflammatory substances discovered that sought to challenge this theory still maintain some association with the release of nitric oxide in the brain (Juhasz et al., 2003).

##### 2.1.2.2 Serotonin

Serotonin, a catecholamine also known as 5-hydroxytryptamine (5-HT), has been linked to migraine pathophysiology and some migraine treatments act on the serotonergic system. In a study conducted by Anthony et al. (1967), they observed an apparent significant fall in the plasma levels of serotonin during a migraine with aura attack (Anthony et al., 1967). Similarly, they observed that reserpine administration led to a fall in serotonin levels and triggered a migraine attack (Anthony et al., 1967). Reserpine is a vesicular monoamine transporter-2 inhibitor that blocks the storage of catecholamines and diminishes their concentrations in the synapse (Wimalasena, 2011). As an intervention strategy exogenous serotonin administration reduced migraine headaches. This might also explain why serotonin agonists and medications that increase serotonin levels and activity in the brain such as tricyclic antidepressants, selective serotonin reuptake inhibitors (SSRIs) and serotonin and norepinephrine reuptake inhibitors (SNRIs) have some beneficial role in the treatment of migraine. Studies have also attempted to examine serotonin metabolism during migraine attacks. In one study, contrary to the initial finding by Anthony et al. (1967), serotonin levels during migraine attacks were observed to be higher as well as an observed decrease in 5-hydroxyindoleacetic acid levels (Anthony et al., 1967). This led the authors to conclude leading to a conclusion that serotonin metabolism is elevated during migraine attacks (Ferrari et al., 1989). This conclusion has been corroborated in a study conducted on migraine patients using positron emitted topography scan monitoring 5-HT_4_ quantification as a surrogate for brain serotonin levels (Deen et al., 2019). In this study, high serotonin levels were detected in correlation with reduced binding to the 5-HT_4_ receptor. This was determined to be due to 5-HT_4_ receptor downregulation, and chronic migraine patients were shown to have high levels of serotonin during migraine attack. Thus, serotonin receptors are likely very important in migraine development, maintenance, and treatment.

###### 2.1.2.2.1 Serotonin receptors

The serotonin receptors 5-HT_1*B*_ and 5-HT_1*D*_ have been strongly implicated as having a critical role in migraine pain (Buzzi and Moskowitz, 1991; Tepper et al., 2002). The 5-HT_1*B*_ receptors mediate vasoconstriction in the cerebral arteries when stimulated by serotonin and its agonists, reversing the vasodilation in arteries to reduce migraine headache. While the 5-HT_1*B*_ vasoconstrictive effect is also seen in coronary arteries and contributes to its cardiovascular hemodynamic effect, the 5-HT_1*D*_ receptor is localized within the brain and is involved in inhibiting nociceptive neuropeptide release from the trigeminal system, and minimal vasoconstrictive effects (Goadsby and Hoskin, 1998; Longmore et al., 2000). When stimulated by serotonin 5-HT_1*B*_/5-HT_1*D*_ receptors also inhibit nociceptive signaling between the spinal cord and the brainstem trigeminocervical complex (Tepper et al., 2002). Essentially, these receptors are involved in terminating migraine headache when stimulated and their discovery has led to the drug class known as triptans.

Another class of serotonin receptor, 5-HT_1*F*_ receptors, are also important in migraine treatment. Unlike the 5-HT_1*B*_ and 5-HT_1*D*_ receptors, agonist activity at 5-HT_1*F*_ receptors does not cause arterial vasoconstriction but instead primarily modulates neurotransmitter release in the brain. The abundance of 5-HT_1*F*_ receptors in the meninges of the brain, hypothalamus, thalamus and cortex, and presence in trigeminal ganglion neuron terminals allows their ability to block the release of calcitonin gene related peptide (CGRP), as well as glutamate transmission when stimulated by serotonin (Clemow et al., 2020). Because 5-HT_1*F*_ agonists (ditans) do not produce vasoconstriction, especially in coronary vessels, they do not have hemodynamic side effects and are an attractive therapeutic alternative to 5-HT_1B/1D_ receptor agonists (triptans). The introduction of the peptide calcitonin gene related peptide in this current discussion leads to another theory that deviates from attributing migraine headache solely to vascular mechanisms but also considers how the brain and some associated peptides contribute to migraine headache.

#### 2.1.3 The neurovascular theory of migraine

##### 2.1.3.1 The role of the trigeminovascular system in migraine

Previous studies have shown that certain parts of the brain play a critical role in the pathophysiology of migraine including the trigeminovascular system (Goadsby and Edvinsson, 1994; Stankewitz et al., 2011). The trigeminal nucleus lies in the medulla of the brain stem, and receives sensory input about touch, nociception and temperature from the trigeminal nerve, the facial nerve, the glossopharyngeal nerve, and the vagus nerve. The brain stem has long been associated with migraine triggers, a finding discovered with the aid of functional imaging techniques (Weiller et al., 1995; Bahra et al., 2001). One study that challenged the vascular theory by implicating the potential role of peptides in migraine was conducted by Goadsby et al. (1990) in which an increase in neuropeptide Y, substance P and CGRP in external jugular blood was observed after stimulating the trigeminal ganglion of humans. In another study using cats and humans, trigeminal ganglion stimulation produced CGRP elevations, a response that was attenuated by the anti-migraine drugs sumatriptan and dihydroergotamine (Goadsby and Edvinsson, 1993). The trigeminal nerve which projects into the meninges of the brain was shown to be involved in the migraine attenuation mechanism of serotonin receptors 5-HT_1*A*_, 5-HT_1*B*_, and 5-HT_1*D*_. Agonists of these receptors (triptans) were able to inhibit the electrophysiologic effect of the trigeminal neurons in an isolated superior sagittal sinus (Goadsby and Classey, 2003). Similarly, although nitric oxide, through vasodilatory mechanisms, is able to induce migraine, in humans nitric oxide may activate the trigeminal system through release of peptides and inflammatory substances such as prostaglandin E_2_ (PGE_2_), neurokinin A, as well as cAMP (Sarchielli et al., 2000). Magnetic resonance imaging has been conducted on patients who received nitroglycerin infusion and although there was a short-lived vasodilation, unilateral headache persisted with minimal change in cerebral arterial blood flow (Schoonman et al., 2008). The persisting headache can therefore not have been due to vasodilation but through other mechanisms that cause sensitization of nociceptors located in the meningeal regions of the brain. In another experiment using functional magnetic resonance imaging of the brain, a cyclical pattern of activity measured as blood oxygenation level-dependent responses (BOLD), was seen after the trigeminal nuclei were stimulated. A strong BOLD response in the trigeminal nuclei upon stimulation predicted an impending migraine attack and this response was highest in the preictal phase which coincides with the prodromal phase of migraine (Stankewitz et al., 2011). These findings demonstrated an acceptance of neurogenic mechanisms that happen in tandem with vasodilation to explain the pathophysiology of migraine thus strengthening evidence on the neurovascular theory of migraine. But the existing knowledge is not entirely absolute because different markers and receptors come up in migraine research findings that additionally contribute to migraine pathogenesis.

##### 2.1.3.2 Calcitonin gene related peptide—A major molecule associated with the neurovascular theory

As mentioned above, increased CGRP appears to be correlative with migraine, and is one of such biomarkers that has taken precedence with therapeutic agents being developed against it. CGRP has two isoforms, CGRP-α and CGRP-β (Zaidi et al., 1990; Kee et al., 2018). CGRP was identified to cause vasodilation in cerebral vessels (McCulloch et al., 1986) and subsequently shown to be a part of peptides that are released into circulation during migraine headache (Goadsby et al., 1990; Lassen et al., 2002). This peptide is a 37-amino acid neuropeptide and shown to increase markedly during migraine attacks as demonstrated in a human model of migraine (Juhasz et al., 2003). It acts on a receptor which is a heterodimer of calcitonin receptor-like receptor (CLR) and receptor activity modify protein 1 (RAMP1) to elicit its effects (Liang et al., 2018). It is released by the trigeminovascular system, is expressed by thin unmyelinated C fibers, and it is suspected that activation of these fibers causes CGRP release. However, the receptors CLR and RAMP1 are found in thick myelinated A-fibers (Eftekhari et al., 2013). CGRP has been implicated in preclinical studies using transgenic mice (nestinJ/hRAMP1) as a mediator of photophobia associated with migraine (Recober et al., 2009). The receptor density of RAMP1 has been shown to correlate with the susceptibility of patients to the development of migraine as well (Zhang Z. et al., 2007). The sex-linked pattern of migraine may also be partially attributed to CGRP. In an experiment in which migraine-like response was induced in male and female Sprague-Dawley rats significantly increased hyperalgesic responses were observed in female rats treated with CGRP compared to male rats (Paige et al., 2022).

##### 2.1.3.3 Other biological molecules and structures linked to the neurovascular theory of migraine

###### 2.1.3.3.1 Pituitary adenylate cyclase-activating peptide (PACAP)

Pituitary adenylate cyclase-activating peptide (PACAP) is another molecule that is suspected to play a role in migraine. It is structurally related to peptide hormones like glucagon, vasoactive intestinal peptide, and secretin (Harmar et al., 2012). It was isolated in a preclinical rat model and exhibited the ability to stimulate adenylate cyclase release from the anterior pituitary cells (Miyata et al., 1989). PACAP is found in two isoforms known as PACAP-38, the 38-amino acid peptide (Miyata et al., 1989) and PACAP-27, a 27-amino acid peptide, both isolated from rat hypothalamus (Miyata et al., 1990). These two peptides have been shown to induce migraine (Syed et al., 2012; Guo et al., 2016; Ghanizada et al., 2020). Three G-protein coupled receptors are involved in the agonist activity of these PACAP isoforms and they are PACAP type 1 (PAC_1_) receptor, VPAC_1_ and VPAC_2_ receptors (Harmar et al., 2012; Syed et al., 2012). Both PACAP isoforms bind to the PACAP type 1 (PAC_1_) receptor to stimulate the activation of the trigeminovascular neurons, a phenomenon that leads to vasodilation of meningeal arteries (Akerman and Goadsby, 2015). It must be noted that the role of PACAP in migraine is of emerging importance, but it has been employed in the development of human models of migraine where infusion of PACAP38 triggers migraine-like headache (Schytz et al., 2009). Beyond vasodilation, it is suspected that PACAP i.e., PACAP38 may also cause mast cell degranulation in the dura mater (Baun et al., 2012). It is hypothesized that although vasoactive intestinal peptide (VIP) shares the receptors VPAC_1_ and VPAC_2_ with PACAP, it is unable to induce robust degranulation, which might explain why it does not cause headache whereas PACAP activity at VPAC_1_ and VPAC_2_ does cause headache (Baun et al., 2012). In addition, sumatriptan, an approved treatment for acute migraine, relieved PACAP38-induced temporal artery dilation and resultant headache (Wienholtz et al., 2021).

###### 2.1.3.3.2 Glial cells

Glial cells are cells found in abundance in the central and peripheral nervous system. In the central nervous system (CNS), the following subtypes are present: astrocytes, oligodendrocytes, NG2-glia and microglia (Ffrench-Constant and Raff, 1986; Kimelberg, 2010; Kimelberg and Nedergaard, 2010; Jäkel and Dimou, 2017; Vila-Pueyo et al., 2023). In the peripheral nervous system (PNS), Schwann cells, satellite glial cells, olfactory ensheathing cells and enteric glia are present (Duncan et al., 1981; Barnett and Riddell, 2004; Rühl et al., 2004; Griffin and Thompson, 2008; Jäkel and Dimou, 2017). Studies have elucidated the role of astrocytes and microglia in the CNS, and satellite cells in the chronification of migraine, CSD initiation and propagation, as well as the symptomology of headache in migraine. Astrocytes are thought to reduce glutamate and potassium ion concentration in the brain using a Na^+^/K^+^ ATPase (NKA) pump as well as the glutamate transporters excitatory amino acid transporter 1 and 2 (EAAT1, also known as GLAST, and EAAT2 also known as GLT), respectively (Duan et al., 1999; Capuani et al., 2016; Lee et al., 2022). Mice with a heterozygous mutation that leads to a partial functional loss of the α2 subunit of NKA pump had reduced potassium and glutamate clearance rates (Capuani et al., 2016). In the same study electrophysiological recordings revealed that the brains of these mutant mice also were more susceptible to CSD than wild type mice (Capuani et al., 2016). In a nitroglycerin-induced chronic migraine model using C57BL/6J mice microglia are activated in a proinflammatory manner, and increased proinflammatory cytokine protein is observed (Colonna and Butovsky, 2017; Yang et al., 2023). When roxadustat is administered a reduction in microglial activation and proinflammatory cytokine protein production is observed in the trigeminal nucleus caudalis, as well as attenuated migraine-like behavior (Yang et al., 2023). Satellite glial cells support neurons via cross-communication in peripheral ganglia and are found localized in the trigeminal ganglion (Messlinger et al., 2020). There is also some scientific evidence that shows the satellite glial cells also produce nitric oxide that may contribute to orofacial pain (Vause and Durham, 2009). Neuronal processes are therefore not the only contributing factors to migraine pathology, but there is an apparent augmentation provided by glial cells which requires further exploration.

###### 2.1.3.3.3 Histamine

Histamine release has also been identified as a migraine headache contributor. A study conducted by Krabbe and Olesen (1980) showed that infusion of histamine to non-headache, migraine and tension-type headache study participants was able to evoke headache responses in all three groups, and was abolished by the administration of an H_1_ receptor blocker mepyramine (Krabbe and Olesen, 1980). In a later study, it was shown that the headache response elicited by histamine, due to cerebral artery endothelium activity, was comparable to that seen after nitric oxide administration (Lassen et al., 1995). In addition, mast cell degranulation, also associated with the neurogenic inflammation theory of migraine, causes histamine release (Levy et al., 2007). Histamine release can be also stimulated via CGRP (Ottosson and Edvinsson, 1997). It is hypothesized that brain meninges contain nociceptors which are able to interact with trigeminal neurons, and this trigeminal neuron interaction leads to nociceptor sensitization, ultimately leading to pain, and mast cells contribute to this signaling cascade by releasing serotonin, prostaglandin I_2_ (PGI_2_) and histamine (Zhang X. C. et al., 2007). As H_1_ and H_2_ receptor antagonism has not been found to produce consistent reductions in migraine headache no antihistamine migraine treatments are currently available (Worm et al., 2019).

**FIGURE 1:**
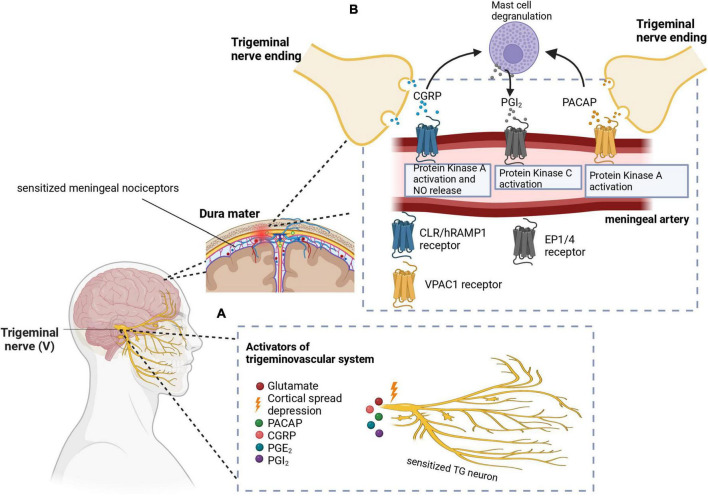
**(A)** Cortical spreading depression (CSD) stimulates the trigeminal nerve. Additionally, other molecules like PACAP, CGRP, PGE2, and PGI2 have been implicated in the activation and sensitization of the trigeminal nerves. **(B)** CGRP and PACAP are released from the nerve endings of the trigeminal nerve to cause vasodilation in the meningeal arteries. Signal transduction mechanisms lead to protein kinase A or C activation, as well as nitric oxide release. Mast cell degranulation also produces inflammatory substances such as PGI2 which leads to sensitization of meningeal nociceptors as well as vasodilation. In these images, PACAP, pituitary adenylate cyclase-activating peptide; CGRP, calcitonin gene-related peptide; PGE2, prostaglandin E2; PGI2, prostaglandin I2; CLR/hRAMP1, calcitonin receptor-like receptor/human receptor activity modifying protein 1 heterodimer; EP1/4, prostaglandin E2 receptor. Adapted from BioRender (2023).

**FIGURE 2:**
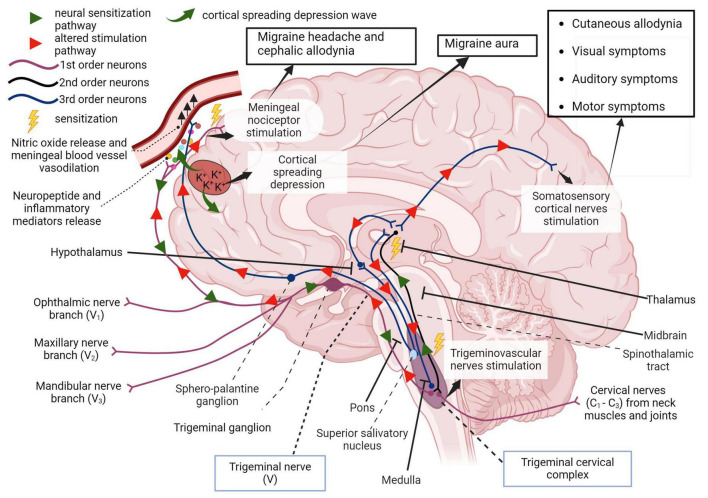
First order afferent nerves of the trigeminal nerve (cranial nerve V) transmit sensory information from the orofacial region and meninges for signal processing in the thalamus. In migraine, peripheral sensitization may occur when neuroinflammatory peptides such as CGRP, as well as processes that lead to mast cell degranulation, cause the downstream sensitization of nerves in the trigeminal pathway. In addition, cortical spreading depression which involves an increase in extracellular potassium ions and enhanced glutamatergic signaling can also trigger this neurogenic response and is also responsible for the symptoms associated with migraine aura. Localized meningeal nociceptor sensitization is another outcome of these processes. The altered afferent signaling pathway sensitizes second order nerves in the trigeminal cervical complex via the trigeminal ganglion and impulses reach the thalamus via the spinothalamic tract. Third order neural projections from the thalamus that innervate the somatosensory cortex, hypothalamus and other areas of the brain undergo an aberrant stimulatory process that causes somatic symptoms of migraine such as extracephalic (cutaneous) allodynia, auditory, visual, and motor impairment. Descending pathways from the thalamus also sensitize the hypothalamus to cause a parasympathetic outflow through the superior salivatory nucleus that promotes nitric oxide release and vasodilation in meningeal blood vessels. The trigeminal nerve endings also secrete neuropeptides that sensitize meningeal nociceptors to cause headache and/or cephalic allodynia (Goadsby et al., 2017; Ashina et al., 2019; Mungoven et al., 2021, 4). Adapted from BioRender (2024).

Graphical representations of the proposed pathophysiology of migraine are shown in Figures 1, 2. In addition, the prevailing theories of migraine are contrasted in Table 2 while a summary of the mediators of migraine are provided in Table 3.

**TABLE 2:** Contrasting the leading theories of migraine pathophysiology.

	Vascular theory	Neurovascular theory	References
Main postulate	Suggests that migraine headache occurs primarily because of vasodilation in the meningeal arteries.	Suggests that migraine headache occurs from an interplay between neuropeptides and neurogenic inflammation	Iversen et al., 1989; Goadsby et al., 1990
Implicated anatomic regions	Meningeal blood vessels	Trigeminal system, meningeal blood vessels, thalamus, hypothalamus	Iversen et al., 1989; Goadsby et al., 1990
Pathological processes	Increase in endogenous nitric oxide production causes an increase in vasodilation in meningeal blood vessels.	The release of peptides such as calcitonin gene related peptide (CGRP), pituitary adenylate cyclase-activating peptide (PACAP), and neuroinflammatory markers such as interleukins, substance P acting as agonist on their receptors, in addition to mast cell degranulation, leads to a cascade of processes that hypersensitize neurons in the trigemino-vascular pathway.	Iversen et al., 1989; Goadsby and Edvinsson, 1994; Sarchielli et al., 2000; Stankewitz et al., 2011; Akerman and Goadsby, 2015
Pharmacologically targeted receptors	Serotonin 5-HT_1B_, 5-HT_1D_, 5-HT_1F_ receptors	CLR/RAMP1 receptor, VPAC1 receptor	Buzzi and Moskowitz, 1991; Tepper et al., 2002; Zhang Z. et al., 2007; Baun et al., 2012; Paige et al., 2022

**TABLE 3:** Summary of mediators in migraine pathophysiology.

	Neuroanatomical location	Function	Migraine theory	References
**Neurotransmitter (receptor)**				
Glutamate (NMDA receptor)	Neurons in trigeminal ganglion, thalamus and trigeminovascular complex	Excitatory neurotransmitter that is elevated in migraine and associated with cortical spreading depression	Neurovascular	Storer and Goadsby, 1999
Serotonin (5-HT_1B_, 5-HT_1D_, 5-HT_1F_)	Trigeminal system, cerebral blood vessels	Low levels of serotonin suspected to trigger migraine; agonist activity of serotonin on receptors causes vasoconstriction of cerebral blood vessels to reduce migraine	Vascular	Buzzi and Moskowitz, 1991; Tepper et al., 2002
**Neuropeptide (receptor)**				
Calcitonin gene related peptide (CLR/RAMP1 receptor)	Trigeminal ganglion and smooth muscles of intracranial blood vessels	Production leads to signaling cascade that causes meningeal vasodilation, as well as sensitization of meningeal nociceptors	Neurovascular	Goadsby et al., 1990; Juhasz et al., 2003
Pituitary adenylate cyclase-activating peptide-38 (PAC_1_, VPAC_1_, and VPAC_2_ receptors)	Sensory nerve fibers in dura mater, brainstem and trigeminovascular system	Causes vasodilation in cerebral blood vessels	Neurovascular	Edvinsson et al., 2018
Histamine (H_1_ receptor)	Mast cell	Released by mast cells to cause meningeal nociceptor sensitization; may also cause vasodilation	Neurovascular	Levy et al., 2007
**Ions**				
Calcium	Intracellular ion in presynaptic terminals and somato-dendritic membranes in brain and spinal cord	Promotes neurotransmitter release at synapses and causes neural excitability; enhanced influx into neurons leads to cortical spreading depression	Neurovascular	Pietrobon, 2013
Potassium	Intracellular ion	Increased efflux causes depolarization and enhances neural excitability; plays a role in the initiation and propagation of cortical spreading depression	Neurovascular	Leao, 1947; Enger et al., 2015; Cozzolino et al., 2018
Magnesium	Intracellular ion	Inhibits the influx of calcium into neurons; low levels in migraine decreases the threshold for neuronal excitability.	Neurovascular	Lodi et al., 2001
Sodium	Extracellular ion	Increased influx into neurons causes depolarization of cells and promotes cortical spreading depression	Neurovascular	Leao, 1947
**Biomolecules**				
Nitric oxide	Endothelium of meningeal and dural blood vessels	Produced by nitric oxide synthase and causes vasodilation of meningeal and dural blood vessels	Vascular	Iversen et al., 1989

##### 2.1.3.4 Sex-linked hormones in migraine pathophysiology

Above, it is mentioned that migraine is prevalent in women, and efforts to understand this phenomenon have required investigating the role of menstrual cycle stage and sex hormones in migraine pathophysiology. In a cohort study of premenopausal women with migraine, it was observed that migraine attacks were frequent in the peri menstrual period (2 days before menstruation up to 3 days after the onset of menstruation) with or without aura (Verhagen et al., 2023). A case control study also showed the use of oral contraceptives predisposed patients who experience migraine with aura to increased migraine headaches, and menstruation triggered migraine in patients who suffered from migraine without aura (Granella et al., 2000). Early studies have shown migraine also occurs in pregnancy, especially in patients who experience migraine with aura (Cupini et al., 1995). The estrogen receptor alpha activation, through estrogen agonist activity, is sufficient to produce endothelial nitric oxide (Chen et al., 1999). In preclinical studies, susceptibility to CSD was found to be increased in rats treated with estradiol. Increased CSD due to estradiol may explain the observed increase in migraine with aura among pregnant patients (Chauvel et al., 2018). Immunohistochemistry results also revealed male and female rats do not appear to display differences in CGRP and PACAP, estrogen receptors alpha and beta were more highly expressed alongside CGRP and PACAP in females within the trigeminal ganglion. In addition, in response to estrogen receptor beta stronger middle cerebral artery endothelial vasodilation was observed in female than male rats (Warfvinge et al., 2020).

#### 2.1.4 Genetics in migraine pathophysiology

Genetics plays a role in migraine development as well, and the ICHD-3 document categorizes a rare type of migraine known as familial hemiplegic migraine (FHM) involving migraine aura with motor weakness with a first or second-degree family member having a similar clinical presentation (Headache Classification Committee of the International Headache Society (IHS), 2018). While this form of migraine is not a focus of this review, it is worth mentioning that mutations in the CACNA1A gene, ATP1A2 gene and SCN1A gene lead to the three forms of FHM, respectively, i.e., FHM1, FHM2 and FHM3 (Tottene et al., 2002; De Fusco et al., 2003; Carreño et al., 2013). Migraine with or without aura has been found to have genetic influences as well. One of the early studies conducted to investigate this influence utilized a population-based survey to find out how many migraine patients had relatives who also had migraine. First degree relatives of patients who suffered from migraine with aura had about four times the risk of developing migraine with aura. In a similar manner, first degree relatives of patients who suffered from migraine without aura also had an increased likelihood to develop either migraine with or without aura (Russell and Olesen, 1995). Likewise, a population-based twin study found some level of genetic influence on migraine without aura among monozygotic and dizygotic twins (Gervil et al., 1999). Also, polymorphism in the serotonin transporter transcription gene (5-HTT gene), which is implicated in anxiety related disorders (Lesch et al., 1996), has been associated with inherited migraine. Anxiety is sometimes experienced with migraine and a clinical study showed there was a higher frequency of the s allele of the 5-HTTLPR genotype present in migraine patients (Gonda et al., 2007). The 5-HTTLPR polymorphic variant has also been seen in migraine with aura patients (Borroni et al., 2005). Another finding characterizes the presence of an X-linked dominant allele present on chromosome Xq24-28 that increases the susceptibility to developing migraine (Nyholt et al., 2000). Additionally, ten molecular markers have been detected in the estrogen receptor 1 gene with three of the haplotypes being found to be linked to migraine (Rodriguez-Acevedo et al., 2013).

### 2.2 United States FDA-approved migraine treatments

#### 2.2.1 Abortive therapies

Migraine treatments fall under two different therapies -abortive or preventive. Abortive medications are taken to stop acute migraines, while preventive medications are taken daily to reduce the occurrence of migraine headaches. A comprehensive review by Zobdeh et al. (2021) classifies all migraine therapies approved by the United States Food and Drug Administration (US FDA) from 1970 to 2020 and a summary is given below. Additionally, in this review newer medications that underwent examination in clinical trials from 2020 to 2023 will be highlighted.

Ergotamine is one of the oldest treatments approved for migraine, but due to its potent vasoconstrictive action its use is hampered by rebound headache as well as a cardiovascular condition contraindication (Tfelt-Hansen et al., 2000). Triptans, serotonin receptor agonists, are the most used drug class used to manage migraines. Some common examples of triptans approved by the US FDA are sumatriptan, zolmitriptan, eletriptan, frovatriptan, naratriptan, rizatriptan, and almotriptan. Zolmitriptan, naratriptan, rizatriptan, almotriptan, frovatriptan are non-selective 5-HT_1*B*_ and 5-HT_1*D*_ receptor agonists (Steiner et al., 2003; Marmura et al., 2015; Zobdeh et al., 2021). Eletriptan is a non-selective 5-HT_1*B*_, 5-HT_1*D*_, and 5-HT_1*F*_ receptor agonist. Despite their common use in clinical practice, they often produce medication overuse headache, and the discontinuation rate of triptans is between 50 and 82% (Yang et al., 2021), One major contraindication to triptan use is 5-HT_1*B*_ mediated vasoconstriction which can negatively impact existing cardiovascular conditions. Ditans, 5-HT_1*F*_ receptor agonists, do not possess the vasoconstrictive activity of triptans, and Lasmiditan is the only member of this class that is FDA approved (Kuca et al., 2018). CGRP receptor antagonists known as gepants are an alternative to triptans and have also been approved to manage acute migraine (Croop et al., 2019). These molecules directly bind to the CLR/RAMP1 receptor to antagonize the pharmacologic effect of CGRP. Rimegepant, ubrogepant, and zavegepant are the current gepants approved by the US FDA for acute migraine treatment (Croop et al., 2019; Dodick et al., 2023; Lipton et al., 2023).

Over-the-counter medications are also used to treat migraine and may be available in combinations. Acetaminophen, caffeine, and non-steroidal anti-inflammatory drugs such as ibuprofen, aspirin, celecoxib are some examples of over-the-counter medicines used for acute migraine (Goldstein et al., 1999, 2006; Zobdeh et al., 2021).

#### 2.2.2 Preventive therapies

For the prevention of migraine, CGRP human monoclonal antibodies have been developed that either bind CGRP and inhibit CGRP binding to its cognate receptor or directly block CGRP receptors. CGRP-related drugs that have been FDA-approved include erenumab (CGRP receptor blocker), galcanezumab, fremanezumab, and eptinezumab (Silberstein et al., 2017; Detke et al., 2018; Lipton et al., 2020). Other drugs that are FDA-approved for migraine prophylaxis include atogepant (CGRP receptor antagonist), onabotulinum toxin A (acetylcholine release inhibitor), topiramate (sodium channel blocker/glutamate antagonist/GABA modulator), amitriptyline (tricyclic antidepressant), valproic sodium, valproic acid, propranolol (non-selective beta blocker) (Diener et al., 2007; Dodick et al., 2009; Pringsheim et al., 2010; Herd et al., 2018; Steiner et al., 2019; Pozo-Rosich et al., 2023). A summary of approved medications is provided in Table 4.

**TABLE 4:** Currently approved migraine therapeutics (adapted from Zobdeh et al., 2021).

Drug	Pharmacologic activity in migraine	Pharmacologic use
**Triptans** Sumatriptan, zolmitriptan, eletriptan, frovatriptan, naratriptan, rizatriptan, almotriptan	Non-selective 5-HT_1B_ and 5-HT_1D_ receptor agonist **Eletriptan also has 5-HT_1F_ agonist activity	Used to treat acute migraine
Lasmiditan	5-HT_1F_ receptor agonist	Used to treat acute migraine
**Gepants** Rimegepant, ubrogepant, zavegepant	CLR/RAMP1 receptor antagonist	Used to treat acute migraine
**Non-steroidal anti-inflammatory drugs** Ibuprofen, aspirin, celecoxib	COX-1/2 receptor antagonist **Celecoxib is a selective COX-2 receptor antagonist	Used to treat acute migraine
Atogepant	CLR/RAMP1 receptor antagonist	Used for migraine prophylaxis
**Monoclonal antibodies** Galcanezumab, fremanezumab, eptinezumab, erenumab	Binds and inhibits CGRP **Erenumab is a CLR/RAMP1 receptor antagonist	Used for migraine prophylaxis
Onabotulinum toxin	Cleavage of SNAP-25 to inhibit the release of acetylcholine	Used for migraine prophylaxis
Topiramate	Sodium channel blocker/NMDA receptor antagonist/GABA modulation	Used for migraine prophylaxis
Amitriptyline	Tricyclic antidepressant	Used for migraine prophylaxis
Propranolol	Non-selective beta-1 and beta-2 receptor antagonist	Used for migraine prophylaxis
Valproic acid	GABA transaminase inhibitor	Used for migraine prophylaxis

**Means the statement is an exception to the initial description provided in the table’s cell.

### 2.3 Novel and experimental therapeutics

Currently, LU AG09222 is an investigational monoclonal antibody that targets PACAP38 and has completed a phase 2 clinical trial to evaluate their use in migraine headache prevention (Rasmussen et al., 2023). Cannabidiol in combination with cannabigerol and tetrahydrocannabinol is in a Phase 4 clinical trial to investigate its use as an adjuvant therapy to medication commonly used to prevent migraine (Kaup, 2023). These clinical trials demonstrate that additional targets may prove to be fruitful to examine and may yield novel migraine therapeutics.

In addition to these, some drugs have been repurposed and investigated in clinical trials to be used as monotherapy or combination therapy for acute and chronic migraine. Examples include lidocaine which is a local anesthetic and indomethacin which is an NSAID (Di Monda et al., 2003; Schwenk et al., 2022). Ketamine (NMDA receptor antagonist) and bupivacaine (local anesthetic) are other repurposed drugs also used to manage acute migraine (Afridi et al., 2013; Cady et al., 2015; Cohen et al., 2018). A summary of experimental therapeutics is provided in Table 5.

**TABLE 5:** Experimental migraine therapeutics.

Drug	Pharmacologic activity	Proposed pharmacologic use	References
**Monoclonal antibody** LU AG09222	Binds and inhibits PACAP38	Migraine prophylaxis	Rasmussen et al., 2023
Cannabidiol/cannabigerol/ tetrahydrocannabinol	CB_1_R and CB_2_R agonist	Adjuvant therapy for migraine prophylaxis	Kaup, 2023
Lidocaine	Sodium channel blocker	Acute migraine treatment	Schwenk et al., 2022
Indomethacin	COX1/2 inhibitor	Acute migraine treatment	Di Monda et al., 2003; Sandrini et al., 2007
Ketamine	Selective NMDA receptor antagonist	Migraine prophylaxis	Afridi et al., 2013; Cohen et al., 2018
Bupivacaine	Sodium channel blocker	Migraine prophylaxis	Cady et al., 2015

## 3 Complications of migraine

### 3.1 Status migrainosus

Status migrainosus is an intractable and severe form of migraine that persists beyond 72 h and may occur with or without an aura (Headache Classification Committee of the International Headache Society (IHS), 2018). To date, there is no established effective treatment for this migraine complication (Iljazi et al., 2020). This condition, while not fatal, is responsible for frequent hospitalizations and increased healthcare costs. A study by Harnod et al. (2018) also highlighted the tendency for suicide among status migrainosus patients (Harnod et al., 2018).

### 3.2 Migrainous infarction and stroke

Migrainous infarction is a rare migraine complication diagnosed as an ischemic infarction, occurs in a patient experiencing migraine with aura that persists for more than 1 h, and the stroke onset coincides with the migraine attack (Headache Classification Committee of the International Headache Society (IHS), 2018). It must be highlighted that ischemic stroke may occur later in a migraine patient and will not be classified as a migrainous infarct because of its onset. However, stroke is also a migraine complication, with early research suggesting a possible link between the two (Murphy, 1955). Some studies have suggested that migraine could serve as a risk for developing ischemic stroke (Henrich and Horwitz, 1989) but with a higher association among women below 45 years of age (Tzourio et al., 1993).

In addition, magnetic resonance imaging studies point to an increased risk of migraine patients developing subclinical brain infarcts (Monteith et al., 2014) which is associated with higher migraine attack frequency (Kruit et al., 2004). There is evidence that points to migraine patients having ischemic white matter abnormalities (WMAs) seen as hyperintense lesions and suspected to be linked to microvascular damage (Ashina et al., 2021). It has been reported that as much as 59% of migraine patients could have white matter abnormalities (Bashir et al., 2013) and the CAMERA-1 study highlighted a higher prevalence of infratentorial cerebellar infarcts in patients with migraine with aura (Kruit et al., 2005). Compared to women without migraine women with migraine without aura had a higher deep white matter hyperintensity volume (Palm-Meinders et al., 2012). However, no link between WMAs and migraine frequency, migraine severity and migraine type has been found, but these changes may explain why migraine patients are at a risk of stroke-related conditions (Palm-Meinders et al., 2012). There are also studies that highlight an association between migraine and developing cardiovascular disease with relatively higher correlation among patients who experience migraine with aura, especially women (Bigal et al., 2010; Kurth et al., 2020; Ng et al., 2022). Some theories proposed to understand why migraines could lead to stroke include the pre-existence of a patent foramen ovale (PFO) in migraine patients (Lechat et al., 1988; West et al., 2018) although some studies do not confirm this association (Garg et al., 2010). PFO is a condition that is congenital and characterized by the presence of an open channel in the septum of the heart’s atrial wall that causes deoxygenated blood to shunt from the right atrium into the left atrium due to failure to close after birth. The foramen ovale is in the region of the fossa ovalis, a valve that closes under an increase in left atrial pressure at birth. This is different from atrial septal defect which is a structural defect in the wall that separates the right and left atrial chambers (Hari et al., 2015). It has also been proposed that CSD in migraine patients leads to stroke and migrainous infarction (Dohmen et al., 2008; Lauritzen et al., 2011). This hypothesis may not be correct, as while there is an apparent increase of stroke related cases among migraine patients, most studies do not demonstrate that treating migraine treatment also leads to reduced stroke events (Sacco and Carolei, 2011).

### 3.3 Depression

Depression is also a recognized migraine complication. In a prospective study conducted to understand the relationship between migraine and major depression, it was found that migraine could cause major depression and vice-versa (Breslau et al., 1994, 2003). Interestingly, this pattern was not observed with other forms of headache. There is some evidence that points to patients who have migraine with aura, especially women, having a higher predisposition to depression, as well as depression with comorbid anxiety, compared to migraine without aura patients (Oedegaard et al., 2006). The mental health complications of migraine also include a tendency for suicide and drug abuse potential (Breslau et al., 1991, 2012) as well as bipolar disorder, panic disorder and agoraphobia (Fasmer and Oedegaard, 2001). Brain activity studies of migraineurs point to an elevated intrinsic brain activity in the left medial prefrontal cortex with a decrease in the right gyrus rectus, observations also seen in patients with depression. Due to the involvement of the right gyrus rectus in emotional regulation, alterations in this region may also underlie the observation that migraine patients have higher concomitant emotional disorder and addiction diagnosis correlations (Ma et al., 2018). Serotonin modulation has also been proposed as a link between depression and migraine because of its role in both disease states (D’Andrea et al., 1989; Meltzer, 1989).

## 4 Human experimental migraine model

### 4.1 Nitroglycerin—The most widely used method of inducing migraine in humans

One of the major challenges in migraine drug discovery is robust translational model development that encompasses all the typical features of acute/chronic migraine with or without aura. While there have been attempts made to produce such a complete model, none exists currently. In humans, one strategy often utilized to induce an experimental migraine is the administration of nitroglycerin at a continuous infusion rate not exceeding 0.5 ug/kg/min to induce predictable migraine headache (Iversen et al., 1989; Onderwater et al., 2021) or as a sublingual tablet (Juhasz et al., 2003). Another is the administration of sildenafil orally which does not provoke vasodilation in the middle cerebral artery but is able to evoke migraine-like headache (Kruuse et al., 2003). However, this model has not been shown to induce migraine aura (Butt et al., 2022). Histamine has also been infused intravenously to induce migraine but is not commonly used to test experimental compound efficacy (Krabbe and Olesen, 1980; Lassen et al., 1995).

#### 4.1.1 Advantages of the nitroglycerin model in humans

The nitroglycerin model is the most widely used method of stimulating migraine-like headaches in humans. Moreover, it can induce the expression of CGRP as occurs naturally in migraineurs (Juhasz et al., 2003), and the headache is effectively abolished by some migraine therapies. For instance, sumatriptan, a 5-HT_1B/1D_ receptor agonist, reduces CGRP levels as well as headache in patients exposed to nitroglycerin (Iversen and Olesen, 1996; Vanmolkot et al., 2006). In addition, other symptoms such as unilateral headache, prodromal symptoms such as yawning, tiredness, neck stiffness (Maniyar et al., 2014), as well as cranial allodynia (Akerman et al., 2019) have been experienced by study participants exposed to nitroglycerin. As there is a high response rate to nitroglycerin administration, another strength of this model is its reproducibility (Sureda-Gibert et al., 2022). There are no reports of nausea or vomiting associated with the nitroglycerin model, although phonophobia and photophobia have been documented in clinical studies (Karsan et al., 2021). In addition to migraine symptoms monitored in this human experimental migraine model, it is also possible using Doppler and positron emission tomography to measure effects on the cerebral blood vessels and blood flow (Tvedskov et al., 2004; Maniyar et al., 2014), as well as to use magnetic resonance angiography to measure blood vessel diameter changes (Schoonman et al., 2008).

#### 4.1.2 Drawbacks of the nitroglycerin model in humans

Despite the above strengths of the human nitroglycerin model, some a notable flaw includes the ability to provoke migraine aura is not consistent, which limits the understanding of how some treatments might affect CSD or any other underlying mechanism involved in migraine aura production (Sureda-Gibert et al., 2022). In addition, some approved migraine treatments are unable to attenuate migraine headache induced by nitroglycerin (Sureda-Gibert et al., 2022). Olcegepant, a CGRP receptor antagonist, was unable to prevent migraine headache induced by nitroglycerin in 13 patients (Tvedskov et al., 2010b). Similarly, propranolol was unable to reduce the headache induced by migraine, although it is hypothesized that this lack of an effect was due to the inability of propranolol to constrict cerebral arteries (Tvedskov et al., 2004). A randomized controlled trial utilizing nitroglycerin infusions given to sixteen healthy students between the ages of 19 to 27 years old, demonstrated that the study subjects developed migraine-like headache but when given zolmitriptan, an approved medication for acute migraine during the nitroglycerin infusion, the headache was not resolved (Tvedskov et al., 2010a). As this study relied upon therapeutic pretreatment, it highlights a flaw associated with other studies that have been performed using this nitroglycerin model. Although potential therapeutic treatments given before nitroglycerin administration demonstrate efficacy, there is a possibility efficacy may not be retained when they are given after nitroglycerin administration. In a real-world scenario, abortive treatments are taken after acute migraine headache, and it is ideal that experimental migraine models mimic this treatment strategy. A summary of the advantages and disadvantages of the human migraine model is given in Table 6.

**TABLE 6:** Contrasting the human and rodent nitroglycerin models.

Human model of migraine	Advantages	References
	CGRP release is inducible in the cerebral region of the nitroglycerin model	Iversen and Olesen, 1996; Juhasz et al., 2003; Vanmolkot et al., 2006
	Classical symptoms of phonophobia and photophobia as well as prodromal symptoms experienced by migraineurs can be replicated in the nitroglycerin model	Maniyar et al., 2014; Akerman et al., 2019; Karsan et al., 2021
	Visualization of vascular changes via magnetic resonance angiography, Doppler and positron emission topography	Tvedskov et al., 2004; Schoonman et al., 2008; Maniyar et al., 2014
	Reproducibility of headaches with the nitroglycerin model	Iversen et al., 1989; Onderwater et al., 2021; Sureda-Gibert et al., 2022
	**Disadvantages**	
	Migraine aura is not consistently provoked with the nitroglycerin model	Christiansen et al., 1999; Sances et al., 2004; Sureda-Gibert et al., 2022
	Limited number of ways to model migraine	Krabbe and Olesen, 1980; Lassen et al., 1995; Butt et al., 2022
	Approved treatments for migraine headache such as propranolol and olcegepant do not abolish headache induced by the nitroglycerin model	Tvedskov et al., 2010a; Sureda-Gibert et al., 2022
	Administration time of investigational therapy in the nitroglycerin model does not mimic clinical use.	Steiner et al., 2003; Marmura et al., 2015; Moye and Pradhan, 2017; Zobdeh et al., 2021
Rodent model of migraine	**Advantages**	
	There are more alternative ways to induce migraine-pain in rodents	Burstein et al., 1998; Lukács et al., 2015
	The molecular mechanisms of CSD and how novel therapies affect it can be studied	Brennan et al., 2007; Cui et al., 2015
	The sex-linked nature of migraine can be studied	Avona et al., 2021; Wang et al., 2022
	Genetically modified rodents help to further the discovery of novel targets for migraine	Mason et al., 2017; Chou et al., 2022
	**Disadvantages**
	Dose of nitroglycerin used to induce migraine in rodents is relatively high compared to humans	Iversen et al., 1989; Akerman et al., 2019; Onderwater et al., 2021
	Invasive procedures involved in direct application into the brain may potentially confound results	Zhao et al., 2017; Wang et al., 2022
	Administration time of investigational therapies does not mimic clinical use	Diener et al., 2007; Pringsheim et al., 2010; Pradhan et al., 2014; Steiner et al., 2019

## 5 Animal migraine models

### 5.1 Nitroglycerin—The most widely used migraine trigger in rodents

Like the human model, algogenic substances that trigger migraine-like headaches have been evaluated on rodents. Nitroglycerin-induced migraine is also a commonly used preclinical experimental model, especially rodents, due to the relative ease in route of administration, usually intraperitoneally (Pradhan et al., 2014; Marone et al., 2018). It may also be administered subcutaneously but this route of administration is not frequently used (Akerman et al., 2019). It also induces peptide and inflammatory marker expression, and has been shown to induce headache-like features that are similar to the temporal nature of migraine, described as a persistent headache (De Logu et al., 2019). Nitroglycerin administration also allows the development of acute and chronic migraine models. A dose of 10 mg/kg nitroglycerin given on alternating days for 9 days (5 doses) in mice as a chronic model of migraine has been shown to induce mechanical allodynia whose effects were suppressed with the administration of topiramate (Pradhan et al., 2014).

One challenge associated with nitroglycerin as a preclinical migraine model is that with this model, the nitroglycerin dose used in animals (i.e., 10 mg/kg), tends to be extremely high compared to the usual nitroglycerin dose administered to humans, approximately 0.5 ug/kg/min for 20 min (10 ug/kg) (Akerman et al., 2019). To address this issue, some studies utilize priming agents that sensitize rodents prior to lower nitroglycerin dose administration (Sureda-Gibert et al., 2022). In addition, some Sprague-Dawley rats are predisposed to lower thresholds of pain and develop spontaneous allodynia at lower (i.e., 0.1 mg/kg) nitroglycerin doses (Oshinsky et al., 2012). Another disadvantage of the preclinical nitroglycerin model is that CSD cannot be reliably induced (Bates et al., 2010) and it is unsuitable for studying the effects of migraine medications on migraine aura.

Most studies utilizing a chronic migraine model often involve the concomitant administration of nitroglycerin with the drug intervention under study, separated by at least 1 h (Pradhan et al., 2014). This chronic preclinical model does not directly parallel the human migraine state where the patient has a basally existing migraine predisposition and migraine attacks occur spontaneously. In addition, migraine therapies are usually clinically given after acute migraine headache or in the case of preventive therapy, daily treatment is given in the migraine patient to reduce future spontaneous migraine occurrence or for newer biologics, monthly or quarterly administration (Diener et al., 2007; Dodick et al., 2009; Pringsheim et al., 2010; Herd et al., 2018; Steiner et al., 2019; Pozo-Rosich et al., 2023). The current chronic migraine nitroglycerin model lacks this spontaneous nature and the seminal experiments utilizing this model showed a return to baseline about 6 days after discontinuing nitroglycerin administration, representing a “loss of chronicity” (Pradhan et al., 2014). In animal care, it is understandable that a state of perpetual nociception might be undesired, but this model is not technically “chronic.” Although such a model has not been developed, an ideal modified preclinical model would be a model that predisposes an animal to spontaneous migraine, coupled with the use of therapeutic preventative treatment administration, which would allow for the exposure of the animal to migraine stimuli, and would determine if the therapeutic reduces migraine headache frequency.

### 5.2 Direct application of inflammatory mediators on brain meninges

Another method employed to induce preclinical migraine model utilizes stereotaxic procedures to penetrate the skull of an alive animal and involves the infusion of substances directly on the dura mater This method allows for the interaction of these inflammatory substances with meningeal nociceptors to elicit a migraine-like response. However, this method to induce migraine-like behaviors is not without caveats. A new migraine model involving direct infusion of a histamine, serotonin, bradykinin, and prostaglandin E_2_ (PGE_2_) combination into the dural space caused periorbital allodynia (Han et al., 2017). However, in this study, the levels of PACAP were decreased rather than increased. While the authors concluded decreased PACAP was representative of decreased PACAP commonly observed clinically during the interictal phase of migraine, it is possible the application method may have been a confounder. A rat study utilizing serotonin and PGE_2_ administration to the dural mater demonstrated these substances diffused into the cerebral cortex and into the cerebrospinal fluid (Zhao et al., 2017). In a similar rat study, using CGRP tagged with fluorescein, which was directly administered to the dura, the florescent marker diffused into nearby regions beyond its site of application in the cerebellum (Wang et al., 2022) and calls into question whether substances applied via stereotaxic surgery evoke responses beyond their interaction with dura mater neurons. Stereotaxic procedures commonly used to apply algogenic substances into the brain involve a level of tissue damage and stress, which may be sufficient to upregulate inflammatory markers (McCluskey et al., 2008).

### 5.3 Behavioral measurements used in rodent migraine models

In animals, behavioral changes, and biomarkers, described below in the next paragraph, that have a clinical correlation to human migraine endpoints are typically examined. Grimacing behaviors in rodents (mice and rats) have been monitored either via manual video scoring or automated via imaging software that is able to capture and score stereotypical headache-like responses, including orbital tightening, whisker changes, ear changes as well as cheek flattening after migraine-inducing agent administration (Langford et al., 2010; Sotocinal et al., 2011; Burgos-Vega et al., 2019; Viero et al., 2022). In rodents spontaneous head rubbing with the paws after migraine-inducing agent administration is also used to examine acute nociception, while allodynic responses including head or hind paw withdrawal responses via the von Frey assay is used as a surrogate for cutaneous allodynia which often occurs in migraine patients (De Logu et al., 2019). The tail flick and Hargreaves tests are used to examine thermal hyperalgesia (Bates et al., 2010; Harriott et al., 2019) which has been characterized in chronic and episodic migraine patients (Schwedt et al., 2011). Similarly, anxiety associated with migraine has been tested with the open field test and light/dark box test (Bates et al., 2010; Wang et al., 2021).

### 5.4 Biomarkers assessed in rodent migraine models

While no specific molecule unique to migraine has thus far been identified, therapeutic drugs suppress the expression of molecules implicated in migraine pathophysiology. Indeed, animal migraine models are able to induce the expression of these molecules, which strengthens the translational and face validity of these preclinical models. CGRP is frequently examined preclinically, due to its clinical overexpression in migraine patients. Immunohistochemistry, immunofluorescence or Western blot studies that utilize CGRP antibodies are often used (Lukács et al., 2015; Zhang et al., 2019). Measurement of the PACAP as well as other inflammatory substances, like PGE_2_ and PGI_2_, interleukin-6 (IL-6), tumor necrosis factor (TNF-α) and the c-Fos gene, all of which are typically upregulated in both clinical and preclinical migraine studies, have also been utilized (Han et al., 2017; De Logu et al., 2019). c-Fos is a proto-oncogene responsible for Fos protein production. It is expressed in the brain and plays multiple functions in brain development (Velazquez et al., 2015), inflammation (Hop et al., 2018) and stress response (Ceccatelli et al., 1989; Senba et al., 1993). It has also been implicated as a marker for pain, as it is expressed in the brain stem, hypothalamus, and spinal cord after noxious stimuli application (Harris, 1998). This gene is usually used as a marker to detect neuronal activation pathways and has aided in the confirmation that the trigeminovascular system is a significant migraine pathology contributor. In a seminal preclinical study, upon activation of the trigeminal system Fos and nitric oxide were both markedly upregulated (Hoskin et al., 1999). In addition, c-Fos expression was found to be elevated in a chronic migraine mouse model and in these mice administration of an approved CGRP antagonist, olcegepant, reduced c-Fos immunostaining in the medial prefrontal cortex and the trigeminal nucleus caudalis (Wu et al., 2022).

### 5.5 Rodent migraine model advantages

#### 5.5.1 Flexibility in testing methods in animal migraine models

Animals are used in translational models within the drug discovery process to understand novel compound pharmacodynamic effects. It is imperative that these models mimic the human condition as closely as possible. In rats, dura mater stimulation via chemical inflammatory substance application as well as selective brain region electrophysiological recordings have led to a greater understanding over the role that brain stem trigeminal neurons, through peripheral sensitization, play in migraine pain (Burstein et al., 1998). Additionally, an “inflammatory soup” containing Complete Freund’s Adjuvant and a cocktail of bradykinin, serotonin, PGE_2_ and histamine, applied to rat dura mater has led to the understanding that meningeal inflammation is a critical migraine pathophysiological modulator (Lukács et al., 2015). The role of neurogenic peptides in migraine has also been successfully elucidated by observing the effects of their administration or antagonism in rodents. For instance, when CGRP was injected into the cerebellum of mice, in predominately female mice it induced migraine-like features such as photophobia, cutaneous allodynia and spontaneous pain (Wang et al., 2022). Similarly, CGRP administered intraperitoneally into rats caused spontaneous pain as well as a squinting response which was attenuated by sumatriptan administration (Rea et al., 2018).

#### 5.5.2 Understanding CSD and migraine therapy effects

Understanding CSD and its mechanisms is possible due to preclinical studies involving potassium chloride (KCl) administration into rodent brains, or through electrophysiological brain recordings (Brennan et al., 2007; Cui et al., 2015). In this preclinical *in vivo* migraine model, CSD can also be viewed visually through calcium imaging as well as by measuring optical intrinsic signals (Charles and Baca, 2013; Schain et al., 2019). In addition, Sprague-Dawley rats repeatedly administered migraine prophylactic medications displayed suppressed KCl-induced changes in dural space action potentials in a dose-dependent manner, but this therapeutic effect was not observed in acutely treated rats (Ayata et al., 2006). This study highlights that CSD likely plays a significant role in migraine and that anti-migraine medications may act through CSD inhibition to produce therapeutic activity.

#### 5.5.3 Modeling migraine triggers in rodents

Migraine triggers such as sound, stress and lack of sleep have been evaluated in mice. A study by Avona et al. (2020) using mice in a restraint stress model, were restrained for 2 h a day for three days and were then administered to sodium nitroprusside, a nitroglycerin preparation, at a low dose (0.1 mg/kg). In non-stressed mice this sodium nitroprusside dose had no effect, but repeatedly stressed mice demonstrated nociceptive responses indicative of migraine induction. Sleep deprivation studies in rats have shown that acute sleep deprivation lowers CSD thresholds (Negro et al., 2020). These studies are important, as the ability to preclinically replicate migraine triggers and to confirm potential clinical human translation is a critical migraine model validation endpoint.

#### 5.5.4 Understanding contributors to the sex-linked nature of migraine

Female-specific clinical responses have also been modeled in animals successfully. In male and female rats administered low dose sodium nitroprusside, CGRP was infused into dura mater, with and in a dose-dependent manner, females exhibited greater headache-like responses than male rats (Avona et al., 2019). In mice, CGRP has also been directly injected into the cerebellum and both anxiety as well as spontaneous pain responses, which included photophobia, was more prominent in females than males (Wang et al., 2022). Prolactin, a female expressed sex-related hormone, has been shown to play a role in migraine. In both mice and rats administered prolactin durally, a prolonged effect of facial hypersensitivity was observed in in female animals only, and meningeal prolactin blockade reduced the migraine-like responses induced by durally administered CGRP (Avona et al., 2021).

#### 5.5.5 Understanding migraine pathophysiology through genetically modified rodents

Genetically modified rodents have been used to understand the pathophysiology of migraine. Mice that were genetically modified to over-express hRAMP1, the receptor for CGRP, were used to deduce that CGRP likely acts through peripheral, not central mechanisms, to cause migraine (Mason et al., 2017). The potential role of the purine receptor P2 × 7 in migraine was determined using P2 × 7 receptor deficient mice, and suggests P2 × 7 antagonists may be viable migraine therapeutics (Gölöncsér and Sperlágh, 2014). Moreover, experimental investigation done using VGluT2-GCaMP6s mice exposed to KCl-induced CSD suggests that the thalamus may play a role in migraine aura production (Fu et al., 2022). VGluT2-GCaMP6s mice were obtained by genetically breeding homozygous Vglut2-ires-Cre mice (JAX stock # 028863) and Cre-dependent Ai96 (RCL-GCaMP6s) mice (JAX stock #028866) from the Jackson laboratory (Fu et al., 2022). The hybrid offspring, Vglut2-GCaMP6s mice, allow the imaging of neuronal changes associated with glutamate release in the brain using a calcium probe (Li et al., 2019; Fu et al., 2022). Protein kinase C-delta gene deficient mice do not demonstrate migraine-like responses after nitroglycerin administration, suggesting protein kinase C—delta neurons in the parabrachial nucleus and central nucleus of the amygdala, may play a role in chronic migraine development (Chou et al., 2022). Thus, preclinical studies using genetically modified animals in biomedical research is an invaluable tool and expanding currently available knockout or overexpression animal lines to encompass additional proteins, receptors, and targets of interest holds great potential in elucidating a more precise understanding of the mechanisms involved in migraine.

## 6 Potential drug development focus areas

### 6.1 Addiction and migraine

Migraine patients are predisposed to a secondary medication overuse headache, which occurs when drugs used to treat headache worsen headache and can also lead to drug-seeking behavior as well as drug abuse (Takahashi et al., 2021). In the America symptoms and treatment (MAST) study conducted in 2017, 15% of 13,649 migraine patients reported analgesic medicine overuse, with a higher prevalence in men, and predominantly involved opioids, triptans, barbiturates as well as ergot alkaloids (Schwedt et al., 2018). While it can easily be inferred that pain persistence may be the predominate driving force behind these patients taking more analgesic medicines, it is not known whether the migrainous state causes alterations in the brain that promotes substance use disorder and drug addiction. An early study by Breslau et al. (1991) showed about two to three fold increase in risk of alcohol abuse, nicotine dependence and illicit drug use among young people who suffered from migraine with or without aura as compared to healthy controls. Identifying the interplay between substance use and migraine disorders could expand the range of drug classes used to treat migraine patients to include therapeutics that lack abuse potential or serve as dual purpose migraine and substance use disorder medications.

### 6.2 Migraine and cannabinoids

Cannabis (*Cannabis sativa*) has been reported to have some beneficial effect in reducing migraine-associated headache (Baron, 2018). One study evaluated cannabis use among migraine patients, and found that patients self-reported more frequent cannabis concentrate were used more frequently due to greater reductions in headache than flower use (Cuttler et al., 2020). Few studies have investigated clinical cannabis use as a migraine therapeutic, but several preclinical studies using various cannabinoids have yielded findings that support cannabinoids as viable migraine therapeutics. Cannabis contains ^Δ9^-tetrahydrocannabidiol, which is a cannabinoid receptor 1 (CB_1_R) and cannabinoid receptor 2 (CB_2_R) partial agonist (Devane et al., 1988; Munro et al., 1993; Ortiz et al., 2022). As WIN 55,212-2 mesylate, a CB_1_R agonist suppressed CSD in rats, it is possible that the CB_1_R may also be involved in the aura pathway (Kazemi et al., 2012). Although cannabis and cannabinoid-related compound medicinal use is restricted in some geographical regions of the world, there is potential for cannabinoid-based therapeutics to become viable inclusions within the pharmacological arsenal for migraine treatment and prevention.

### 6.3 Other serotonin receptors in migraine

The 5-HT_7_ receptor is another serotonin receptor found in the spinal cord and is involved in descending pain pathway modulation (Rocha-González et al., 2005). In a rat model 5-HT_7_ receptor agonist activity produced antinociception, and deletion of this receptor produced reduced opioid-induced antinociception (Yanarates et al., 2010). While this study highlights the potential for 5-HT_7_ receptor agonists maybe useful analgesics, 5-HT_7_ receptor antagonism may be more therapeutically advantageous as a migraine therapeutic. In addition, 5-HT_7_ receptors have been identified in the trigeminal ganglion, cortex, and thalamus where it is postulated to be involved in CGRP release, as well as serotonin-induced meningeal artery vasodilation (Terrón and Martínez-García, 2007). In an experimental migraine model using male Sprague-Dawley rats, SB269970, a 5-HT_7_ receptor antagonist, partially decreased trigeminal ganglion mediated CGRP release (Wang et al., 2010). Currently, the study of this receptor is restricted to experimental preclinical migraine models and further human clinical migraine research is needed.

### 6.4 Purinergic receptors in migraine

Purinergic receptors are activated by adenosine or its nucleotides, adenosine diphosphate (ADP), adenosine triphosphate (ATP) and comprise part of the non-adrenergic non-cholinergic system (Burnstock, 2007; Ghiringhelli et al., 2009). While adenosine binds the P1 receptor family, both ATP and ADP bind, with varying affinity, to the P2Y receptor family. For example, ADP primarily stimulates P2Y_1_, P2Y_6_, and P2Y_12_, while ATP stimulates P2Y_2_ and P2Y_4_. For a detailed review over purinergic receptors and neurotransmission, see the review article by Burnstock (2007). Evidence suggests there may be a purinergic role in migraine headache development. In study involving the mouse nitroglycerin chronic migraine model P2Y_12_ receptor upregulation was observed, and both MRS2395 and clopidogrel, P2Y_12_ receptor antagonists reduced CGRP and c-Fos expression in the trigeminal nucleus as well as decreased periorbital allodynia (Jing et al., 2019). While therapeutic targets of the P2Y_12_ receptor might be desirable, drug antiplatelet activity is often associated with risk of bleeding or decreased anticoagulation with prolonged therapy, which can lead to treatment discontinuation (Becker et al., 2011). In the mouse nitroglycerin-induced chronic migraine model the P2X_4_ receptor, another purinergic receptor subtype was upregulated in the trigeminal nucleus caudalis, and antagonism with a P2X_4_ receptor antagonist, 5-BDBD, reduced its expression (Long et al., 2018). This antagonist also reduced CGRP and c-Fos expression as well as decreased cutaneous and periorbital allodynia. While these purinergic receptors may be promising targets, their widespread bodily distribution warrants selective identification of receptor types that are migraine pathogenesis specific.

### 6.5 Transient receptor potential (TRP) channels in migraine

The sensory perception of noxious temperature and chemical stimuli in peripheral tissue has been attributed to a group of cation channels called transient receptor potential channels (Caterina et al., 1997; McKemy et al., 2002; Story et al., 2003; Jordt et al., 2004; Bautista et al., 2005). The transient receptor potential vanilloid 1 (TRPV1) channel was first found to mediate the hot sensation of chili peppers due to the agonist activity of capsaicin, a vallinyl compound, on the channel. Capsaicin activates the TRPV1 channel to open to allow the influx of sodium and calcium ions, which leads to depolarization of nociceptors and the sensation of heat (Caterina et al., 1997; Yang and Zheng, 2017). The TRP family of channels behave similarly by allowing the influx of cations although they differ in their distribution in the body, physiology, and molecular mechanisms (Liedtke et al., 2000; McKemy et al., 2002; Moran, 2018). To date, the following superfamily of TRP channels have been isolated and characterized: TRP ankyrin (TRPA), TRP canonical (TRPC), TRP melastatin (TRPM), TRP mucolipin (TRPML), TRP polycystin (TRPP) and TRP vanilloid (TRPV) (Zheng, 2013; Moran, 2018). There is now emerging evidence that supports the role of TRP channels in migraine pathophysiology due to functional roles associated with their presence on neurons as well as astrocytes within the brain (Wilkerson et al., 2022). Some clinical evidence that highlighted the possible role of TRP channels was obtained when a study conducted on 555 patients found that single nucleotide polymorphisms in TRPV3 and TRPV1 correlated with susceptibility to migraine (Carreño et al., 2012). Conversely, an experiment conducted in male Sprague-Dawley rats revealed the presence of TRPA1 and TRPM8 in the trigeminal ganglion with a higher neuronal expression of TRPM8 in this region (Kobayashi et al., 2005). A limitation of this study is the exclusion of female rats and considering the sex difference that exist in migraine pathophysiology, it is difficult to accept the result of this study as generalizable. It has also been observed in preclinical study that TRPA1 agonist activity leads to the release of CGRP in the trigeminal region (Demartini et al., 2017). In this study, a hemisected preparation of adult male Wistar rat and C57BL/6 wild-type mouse skull was used. TRPA1 and TRPV1 knock-out mice on a C57BL/6 genetic background were also included. Acrolein and mustard oil, both of which have receptor agonist activity on TRPV1 and TRPA1 channels, dose-relatedly induced CGRP release in wild type animals only (Demartini et al., 2017). It is suspected that while TRPA1 channels may play a role in migraine pain, it does not directly activate nociceptors in the meninges to cause migraine and only has a modulatory role (Benemei et al., 2010; Edelmayer et al., 2012; Demartini et al., 2017). Parthenolide is a partial agonist at the TRPA1 channel, desensitizes the channel (Materazzi et al., 2013), and is an active constituent of the feverfew plant, a natural remedy used for headaches (Cady et al., 2011). Although there are no approved migraine therapeutics that act via TRP channels, parthenolide was found to reduce the release of CGRP and associated meningeal vasodilation in adult rats (Materazzi et al., 2013). While it is unclear whether agonist or antagonist activity of TRP channels will be most beneficial in treating migraines, elucidating the role of TRP channels in migraine pathology remains worth pursuing.

## 7 Conclusion

Despite the remarkable advances made to address and reduce the burden of migraine headache on the global population, there is still the need to identify novel migraine therapeutics. Ideally these therapeutics would target receptors and ligands with ideal selectivity, that demonstrate efficacy in preventing and treating migraine headaches and possess minimal side effects. Elucidation of the complex mechanisms that underly migraines would add much needed insight into therapeutic migraine drug development. Although a full understanding of the underlying migraine etiology remains elusive, novel drug targets have emerged which may yield promising therapeutics. The endocannabinoid system, including cannabinoid receptors, purinergic receptors, TRP channels as well as less investigated serotonin receptor subtypes are potential targets that can be considered for new drug therapies. In addition to CGRP, advances being made toward developing biomolecules that target the pituitary adenylate cyclase-activating peptide and its receptors are laudable. Other 5-HT_1F_ receptor agonists are needed to provide therapeutic options in addition to Lasmiditan, and it is possible that structural modifications of these new drugs that either enhance potency or receptor binding will maximize the ditan drug class efficacy. Substance use disorders and addiction may be an overlooked migraine component. This is an especially understudied area, that demands increased focus, especially given the high risk associated with migraineurs to develop medication overuse headaches. Studies that focus on reducing novel migraine therapeutic abuse potential, as well as studying dual purpose analgesic and substance use disorder medications would also be therapeutically advantageous. These novel therapeutics may encourage treatment intervention which would also reduce substance use disorders and its associated risks in this marginalized patient group.

## Author contributions

KFM: Conceptualization, Data curation, Writing – original draft. YTO: Writing – review & editing. LRM: Supervision, Writing – review & editing. JLW: Funding acquisition, Supervision, Writing – review & editing.
